# T50. SYMPTOMATIC AND FUNCTIONAL RESPONSE TO BREXPIP RAZOLE TREATMENT IN PATIENTS WITH ACUTE SCHIZOPHRENIA BY AGE

**DOI:** 10.1093/schbul/sby016.326

**Published:** 2018-04-01

**Authors:** Catherine Weiss, Erin MacKenzie, Francois Therrien, Peter Zhang, Stine Meehan

**Affiliations:** 1 Otsuka Pharmaceutical Development & Commercialization, Inc; 2 Lundbeck Canada, Inc; 3 H. Lundbeck A/S

## Abstract

**Background:**

Atypical antipsychotics are the mainstay of treatment for schizophrenia, and have a meaningful effect on positive symptoms and agitation/aggression. More recently, treatment goals have shifted to target functioning; a cycle of deterioration often occurs in early schizophrenia in which recurring relapse results in decreased functioning.

Brexpiprazole is a serotonin-dopamine activity modulator that is a partial agonist at 5-HT1A and dopamine D2 receptors, and an antagonist at 5-HT2A and noradrenaline alpha1B/2C receptors, all at subnanomolar potency. The efficacy of brexpiprazole has been shown in both short- and long-term studies. In this post-hoc analysis from three short-term studies, the proportion of patients achieving symptomatic and functional response was assessed, grouped by age at baseline.

**Methods:**

Efficacy and functioning data were pooled from three 6-week, double-blind, placebo-controlled studies in hospitalized patients with acute exacerbation of schizophrenia (Vector [NCT01396421]; Beacon [NCT01393613]; and Lighthouse [NCT01810380]), and stratified according to age at baseline (18–35 years; and 36–65 years). For the current analyses, response was defined as reduction in PANSS score of ≥30% from baseline; a CGI-I score of 1 or 2 (much improved or improved); or reduction in PANSS score of ≥30% OR CGI-I score of 1 or 2. Functional response was defined as an increase in PSP total score of at least 10 points. The analyses were conducted using a mixed-model repeated measures (MMRM) approach with all brexpiprazole doses pooled (2-4mg/day).

**Results:**

557 patients aged 18–35 years and 857 patients aged 36–65 years were analysed. For patients aged 18–35 years, a statistically significantly greater proportion of brexpiprazole-treated vs placebo-treated patients had symptomatic response after 6 weeks of treatment (PANSS ≥30%: 40.5% vs 28.7%, p<0.01; CGI-I 1 or 2: 39.9% vs 25.4%, p<0.001; PANSS ≥30% OR CGI-I 1 or 2: 46.2% vs 32.3%, p<0.01). Similar results were observed for patients aged 36–65 years (PANSS ≥30%: 48.7% vs 37.6%, p<0.01; CGI-I 1 or 2: 47.1% vs 32.7%, p<0.0001; PANSS ≥30% OR CGI-I 1 or 2: 54.8% vs 41.6%, p<0.001). For patients aged 18–35 years, a statistically significantly greater proportion of brexpiprazole-treated vs placebo-treated patients had functional response after 6 weeks of treatment (PSP 10 points change: 46.3% vs 33.0%, p<0.01); similar results were observed for patients aged 36–65 years (49.2% vs 38.2%, p<0.01).

The proportion of patients meeting both symptomatic (using ≥30% PANSS improvement or CGI-I score of 1 or 2) and functional response was statistically significantly greater in brexpiprazole-treated patients vs placebo-treated patients regardless of the age group (18–35 years: 37.4% vs 25.4%, p<0.01; 36–65 years: 41.8% vs 30.2%, p=0.01).

**Discussion:**

The results of these analyses confirm that 6 weeks of treatment with brexpiprazole results in symptomatic and functional response in acutely ill schizophrenia patients in both younger patients (age 18 to 35 years) as well as older patients (age 36–65).

